# Inducible Mega-Mediated Macrolide Resistance Confers Heteroresistance in Streptococcus pneumoniae

**DOI:** 10.1128/aac.01319-22

**Published:** 2023-02-27

**Authors:** Sarah Lohsen, David S. Stephens

**Affiliations:** a Departments of Medicine, Emory University School of Medicine, Atlanta, Georgia, USA; b Departments of Microbiology and Immunology, Emory University School of Medicine, Atlanta, Georgia, USA

**Keywords:** *Streptococcus pneumoniae*, pneumococcus, macrolide resistance, heteroresistance, Mega, *mef*(E)/*mel*, inducible resistance

## Abstract

In Streptococcus pneumoniae (*Spn*), the 5.4 to 5.5 kb Macrolide Genetic Assembly (Mega) encodes an efflux pump (Mef[E]) and a ribosomal protection protein (Mel) conferring antibiotic resistance to commonly used macrolides in clinical isolates. We found the macrolide-inducible Mega operon provides heteroresistance (more than 8-fold range in MICs) to 14- and 15-membered ring macrolides. Heteroresistance is commonly missed during traditional clinical resistance screens but is highly concerning as resistant subpopulations can persist despite treatment. *Spn* strains containing the Mega element were screened via Etesting and population analysis profiling (PAP). All Mega-containing *Spn* strains screened displayed heteroresistance by PAP. The heteroresistance phenotype was linked to the mRNA expression of the *mef*(E)/*mel* operon of the Mega element. Macrolide induction uniformly increased Mega operon mRNA expression across the population, and heteroresistance was eliminated. A deletion of the 5′ regulatory region of the Mega operon results in a mutant deficient in induction as well as in heteroresistance. The *mef*(E)*L* leader peptide sequence of the 5′ regulatory region was required for induction and heteroresistance. Treatment with a noninducing 16-membered ring macrolide antibiotic did not induce the *mef*(E)/*mel* operon or eliminate the heteroresistance phenotype. Thus, inducibility of the Mega element by 14- and 15-membered macrolides and heteroresistance are linked in *Spn.* The stochastic variation in *mef*(E)/*mel* expression in a *Spn* population containing Mega provides the basis for heteroresistance.

## INTRODUCTION

Streptococcus pneumoniae (*Spn*) is an opportunistic pathogen and an obligate commensal of the human nasopharynx (NP). *Spn* is commonly present in the NP of young children, with carriage rates in the United States in the post PCV7/13 vaccine era of 32% in children under five (1), and less commonly in older adults, with carriage rates as low as 1.8% in adults over 65 (2). *Spn* is a common cause of noninvasive acute infections such as otitis media, bronchitis, and sinusitis, as well as invasive infections such as sepsis, pneumonia, and meningitis. Historical widespread treatment of *Spn* infections with macrolides has led to the development of high rates of macrolide resistance (3). Despite this, macrolides are extensively used and remain a treatment of choice in cases of outpatient-treated community-acquired pneumonia caused by *Spn* (4, 5). Thus, understanding the mechanisms by which *Spn* manifests resistance to macrolides is important to better guide treatment options.

Resistance to macrolides in *Spn* can be due to three main mechanisms (6). Rarely, ribosomal mutations lead to very high-level constitutive resistance to macrolides-erythromycin MICs >256 μg/mL. More commonly, ribosomal methylation by enzymes such as the *erm*(B) methylase also lead to very high resistance, MICs >256 μg/mL, either in an inducible or constitutive manner. A third important mechanism is provided by the macrolide efflux genetic assembly or Mega element (7). This 5.4 to 5.5 kb genetic element contains two macrolide resistance genes, *mef*(E), encoding an efflux pump, and *mel*, encoding a ribosomal protection protein. In *Spn*, Mega has two alleles, Mega-1 and Mega-2, distinguished by a 100-bp intergenic region sequence difference between *mef*(E) and *mel*, and can be inserted in multiple unique locations in the pneumococcal genome (I, II, III, IV, and V). Our group has previously shown that the Mega element can confer a wide range of resistances to macrolides-erythromycin MICs 2 to 96 μg/mL, encompassing MICs <16 μg/mL and clinically high-level MICs >16 μg/mL (8, 9). Macrolide resistance associated with the Mega element has the M phenotype, providing resistance to 14- and 15-membered macrolides but not to the functionally similar lincosamides and streptogramin B (10). This resistance is increased greatly upon induction with subinhibitory concentrations of mostly 14- or 15-membered macrolides (11). The induction of *mef*(E)/*mel* is similar to the transcriptional attenuation of the *erm*(K) methylase gene and *tet*(M) gene, where antibiotic-bound ribosomes stall during translation of a leader peptide upstream of the structural gene, allowing formation of an anti-attenuator structure and transcription to occur (8, 12).

Most antibiotic resistance in *Spn*, including macrolide resistance, has been thought to follow traditional resistance patterns, with the population of a *Spn* strain susceptible to a narrow range of concentrations (MICs) of the target antibiotic (6). There are several instances, however, of heteroresistant populations of *Spn* against certain classes of antibiotics. These bacteria contain subpopulations that are resistant to concentrations of antibiotic that are more than 8-fold higher than the concentrations that can inhibit the bulk of the population. Documented heteroresistance for which the mechanisms have been investigated in *Spn* has been seen in resistance against certain beta-lactams, cephalosporins, and fosfomycin (13 to 16). Strains (4/9 clinical isolates and 7/16 reference strains) queried by the Muhlemann group in 2007 were found to be heteroresistant to penicillin, and the presence of the Penicillin-Binding Protein 2× variant has been shown to cause heteroresistance to penicillin (13, 14). Heteroresistance to fosfomycin has been associated with the presence of the *murA1* gene, though this gene is not the sole contributor to heteroresistance in *Spn* (15). Epigenetic inheritance of factors that influence cell morphology have been thought to contribute to heteroresistance to the cephalosporin cephalexin (16).

Initial work examining heteroresistant bacteria postulated that resistance of very small subpopulations is due to fixed mutations in a population at some small rate (17). More recent work has shown that perhaps more than half of the cases of heteroresistance in Escherichia coli, Klebsiella pneumoniae, and Acinetobacter baumannii are due to unstable spontaneous tandem amplifications, especially of genes known to be involved in resistance pathways (18). Other factors thought to contribute to heteroresistance, as seen in Klebsiella pneumoniae, are the overexpression of resistance genes or regulatory proteins that act on resistance genes, such as in the case of eravacycline (one of the classes of tetracycline antibiotics) heteroresistance (19). Stochastic gene expression has been proposed as a possible mechanism for transient resistance and possible heteroresistance (18).

Low-frequency but highly resistant subpopulations of bacteria are clinically concerning as they will not appear in traditional resistance screens and may lead to treatment failures. Macrolide treatment of *Spn* infections due to strains demonstrating the M phenotype has resulted in treatment failures (20, 21), and this could in part be a result of the emergence of high-level macrolide resistance (9).

This report shows that all Mega-containing isolates of *Spn* screened display heteroresistance when unexposed to antibiotic, independent of insertion class, and that this heteroresistance is not due to a stable or semistable phenomenon such as mutation or gene duplication. The heteroresistance is eliminated when the *mef*(E)/*mel* genes are induced. By eliminating inducibility of the *mef*(E)/*mel* genes by removal of the 5′ regulatory region, heteroresistance was eliminated. By restoring the *mef*(E)*L* sequence of the 5′ regulatory region, and thus inducibility of the system, heteroresistance was restored, thus linking inducibility of this system with heteroresistance. Thus, stochastic fluctuations in *mef*(E)/*mel* gene expression lead to subpopulations of higher-level-resistant bacteria, and thus confer a heteroresistant phenotype to all Mega-containing *Spn* isolates.

## RESULTS

### *mef*(E) and *mel* mRNA expression levels are correlated with erythromycin MIC values.

**FIG 1:**
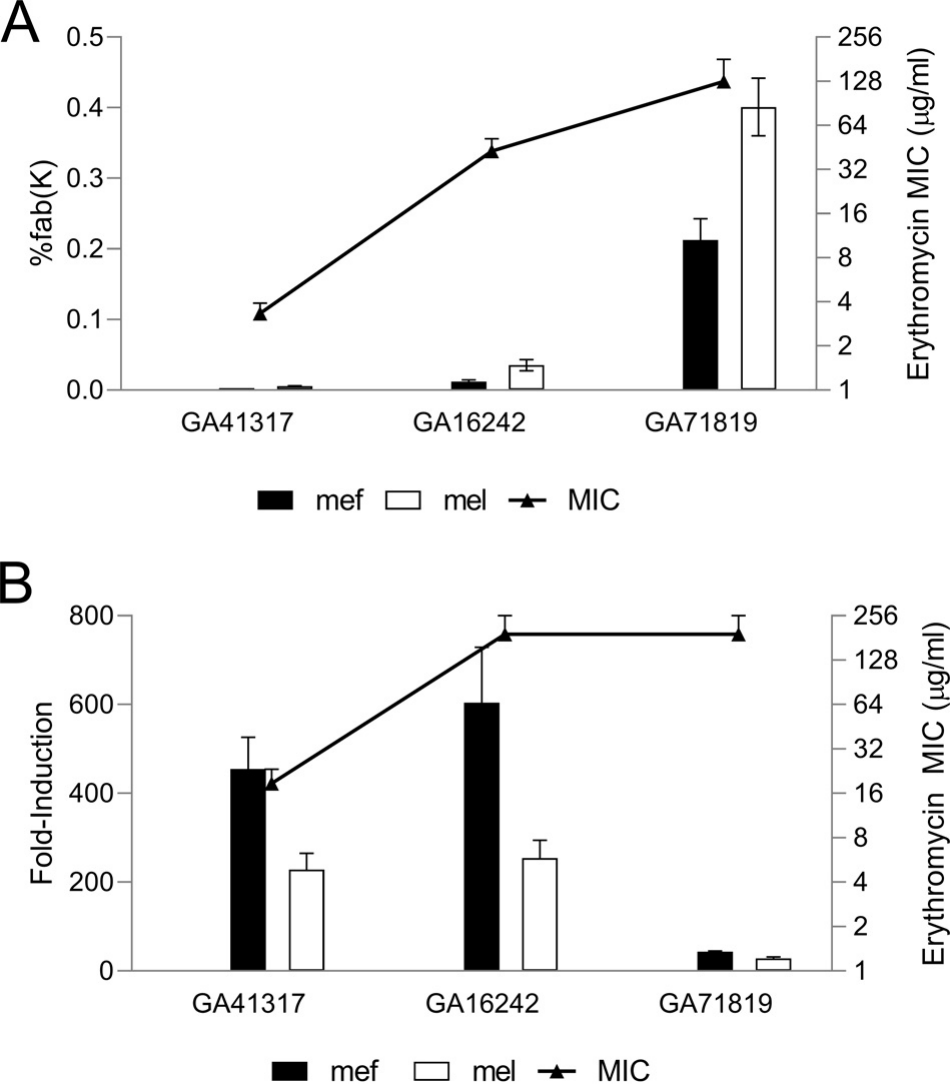
*mef*(E) and *mel* expression levels correlate with erythromycin MIC values. (A) *mef*(E) and *mel* mRNA expression levels and MICs were determined for the lower-level resistant strain GA41317 (erythromycin MIC 3 μg/mL) and the higher-level resistant strains GA16242 (MIC 32 μg/mL) and GA71819 (MIC 64 μg/mL). Expression levels of *mef*(E) and *mel* were plotted as a percentage of *fab*(K). (B) Low- and high-level resistant strains GA41317, GA16242, and GA71819 were induced with subinhibitory concentrations of erythromycin before mRNA expression levels and MICs were determined. *mef*(E) and *mel* expression is plotted as fold over uninduced values. Etesting was performed in at least triplicate to obtain erythromycin MIC values.

Gene expression levels of *mef*(E) and *mel* in Mega containing *Spn* strains with a range of baseline erythromycin MICs were examined. Resistant *Spn* strains GA41317 (erythromycin MIC 3 to 4 μg/mL), GA16242 (MIC 32 to 48 μg/mL), and GA71819 (MIC 64 to 192 μg/mL) were examined. Levels of *mef*(E) and *mel* expression correlated with MICs (Fig. 1A). The very high-level resistant strain GA71819 demonstrated very high intrinsic erythromycin MICs and corresponding high *mef*(E) and *mel* expression levels. In contrast, the low-level resistant *Spn* strain GA41317 demonstrated the lower erythromycin MICs and low *mef*(E) and *mel* expression levels (Fig. 1A), while strain GA16242 was in between.

In previous studies, induction of a low-level resistant strain, GA17457 (MIC 6 to 12 μg/mL), found a 15-fold increase in *mef*(E) and *mel* expression using subinhibitory concentrations of erythromycin, and this increase in expression correlated with increased resistance to erythromycin (11). To determine if similar induction occurred in other strains with various starting MIC values, 1/10th MIC erythromycin was used to induce the strains GA41317, GA16242, and GA71819. Figure 1B demonstrates a 250-fold induction over uninduced for *mef*(E) and *mel* in strain GA41317, and this correlated with a 6-fold increase in MICs. Similar results were seen with the induction of *mef*(E) and *mel* expression in strain GA16242 (Fig. 1B) and a 4.5-fold increase in MICs. The exception was strain GA71819, which was already at very high levels of intrinsic *mef*(E) and *mel* expression that did not change significantly with induction and did not result in a significant change in MIC.

### Mega-containing *Spn* strains display heteroresistant colonies with Etesting.

**FIG 2:**
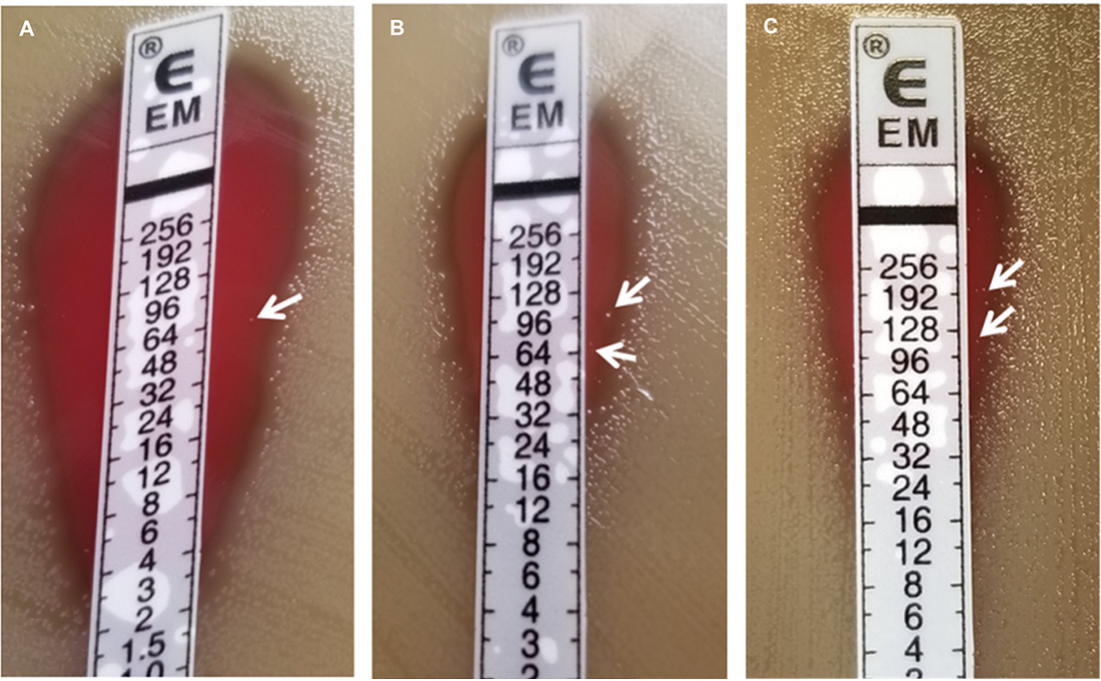
Mega-containing *Spn* isolates display heteroresistant colonies with Etesting. (A) Low-level resistant strain (MIC 3 μg/mL) GA41317, (B) high-level resistant strain (MIC 32 μg/mL) GA16242, and (C) high-level resistant strain (MIC 64 μg/mL) GA71819. White arrows indicate colonies within the zone of inhibition.

In screens of Mega-containing *Spn* clinical isolates to determine erythromycin MICs by Etests, distinct colonies growing in the zone of inhibition of strains GA41317 (Fig. 2A), GA16242 (Fig. 2B), and GA71819 (Fig. 2C) were observed, suggesting heteroresistance to macrolides. To determine stability of the phenotype, colonies were picked from the Etest zone of inhibition in the high-level resistant strain, GA71819, streaked on blood agar plates without selection overnight, and Etesting was repeated. The repeat MICs were consistent with the parent strain (Table 1), indicating the growth of colonies in the zone of inhibition was not due to a fixed mutation or spontaneous tandem duplication event.

**TABLE 1:** Heteroresistant colonies have MICs identical to the parent strain^*a*^

Strain	MIC^*b*^
GA71819	96-192
GA71819 het^R^ a	96-128
GA71819 het^R^ b	96-128

a Heteroresistant colonies were picked from Etest plates, passaged once on TSAII plates without antibiotic, and then subjected to Etesting.

b MICs determined by Etest in at least triplicate and reported as μg/mL. Ranges provided when replicates varied.

To look for gene amplifications, GA71819 was grown in the presence of 1/10th MIC and high levels of antibiotic (192 μg/mL) to select for heteroresistant subpopulations. Genomic DNA was isolated from these cultures. Querying this DNA for copy number of *mef*(E) and *mel* revealed that there was no increase in gene copy number (Table 2). Similar results were obtained for the lower-level resistant GA17457 strain (data not shown).

**TABLE 2:** Copy number of *mef*(E) and *mel* genes relative to *fab*(K) control remain unchanged when selecting for heteroresistant populations

Strain	Relative copy no. *fab*(*K*)	(mean [SD]) *mef*(E)	of gene: *mel*
GA71819 untreated	1.00 (0.00)	1.13 (0.03)	0.92 (0.10)
GA71819 14.4 μg/mL ERY	1.00 (0.00)	1.28 (0.19)	0.96 (0.11)
GA71819 192 μg/mL ERY	1.00 (0.00)	0.95 (0.14)	0.88 (0.08)

### Mega-1 and Mega-2 genotypes in five different insertion sites all display heteroresistance.

**FIG 3:**
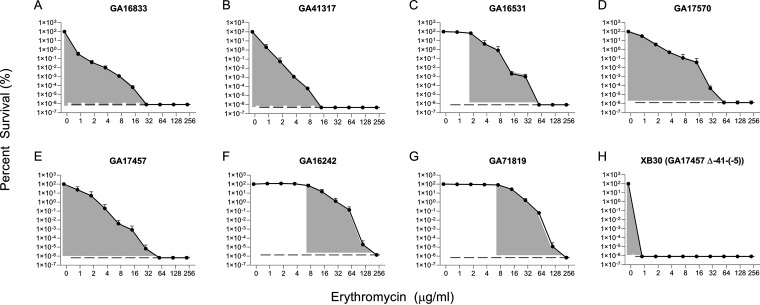
Mega-containing *Spn* isolates all display heteroresistance by PAP analysis. Erythromycin PAP analyses for (A) GA16833, (B) GA41317, (C) GA16531, (D) GA17570, (E) GA17457, (F) GA16242, (G) GA71819, and (H) XB30 (GA17457 (Δ-41-(-5))). MIC data are shown in Table 3. The lower limit of detection is indicated on each graph with a dashed dark gray line. Shaded gray areas indicate the region from the highest noninhibitory concentration to the lowest concentration exhibiting maximal inhibition.

To determine if *Spn* strains containing the Mega element in the five most common insertion sites displayed heteroresistance, population analysis profiling (PAP) was performed. The *Spn* strains chosen represented both Mega-1 and Mega-2, five insertion sites, as well as those displaying both high and low levels of macrolide resistance (Table 3). All strains that contained Mega screened by PAP analysis displayed heteroresistance as defined by a greater than 8-fold difference between the lowest concentration of erythromycin exhibiting maximum inhibition and the highest noninhibitory concentration (Fig. 3A to F). To verify that the expression of *mef*(E)/*mel* from the Mega element was necessary for heteroresistance, XB30, a mutant which does not express *mef*(E)/*mel* due to a deletion of the transcriptional start site, was screened and found to be completely sensitive to erythromycin (Fig. 3G). To further quantitate heteroresistance, the area under the curve (AUC) and the proportion of the curve falling beyond the MIC breakpoints (HR-AUC), shown in the gray shadings (Fig. 3), was plotted for the heteroresistant strains determined by PAP (Fig. 3, Table 4). HR-AUC values ranged from 21.31 to 34.39 (X = 25.95) and represented 42% to 100% of the total AUC. The XB30 erythromycin sensitive strain AUC was 4.04.

**TABLE 3:** *Spn* isolates with MICs to erythromycin and Mega genetic insertion sites

Strain	MIC^*a*^	iMIC^*a*^^,^^*b*^	Mega	Source
GA16833	2 to 3	16 to 32	Mega-1.V	Chancey et al., 2015 (22)
GA41317	3 to 4	16 to 24	Mega-1.IVb	Chancey et al., 2015 (22)
GA16531	6	24 to 32	Mega-2.II	Chancey et al., 2015 (22)
GA17570	6 to 12	32 to 64	Mega-1.III	Chancey et al., 2015 (22)
GA17457	6 to 12	24	Mega-1.I	Zahner et al., 2010 (32)
GA17457 (Δ-41-(-5)) XB30	0.19 to 0.25	ND	Mega-1.I (Δ-41-(-5))	Chancey et al., 2015 (22)
GA17457 (Δ+19-298) XB31	8 to 12	16	Mega-1.I (Δ+19-298)	Chancey et al., 2015 (22)
GA17457 (Δ+63-298) XB38	24	128	Mega-1.I (Δ+63-298)	Chancey et al., 2015 (22)
GA16242	32 to 48	128 to 256	Mega-2.IVa	Chancey et al., 2015 (22)
GA71819	64 to 192	128 to 256	Mega-2.II	Schroeder et al., 2019 (9)

a MICs determined by Etest in at least duplicate and reported as μg/mL. Ranges provided when replicates varied.

b Induced MIC values, with 0.1 μg/mL subinhibitory erythromycin concentration used for induction.

**TABLE 4:** Areas under the curve (AUCs) for uninduced and induced *mef*(E)/*mel* containing wild-type strains

Wildtype uninduced	GA16833	GA41317	GA16531	GA17570	GA17457	GA16242	GA71819
AUC	23.5	21.31	39.42	34.39	31.94	54.5	56.7
HR-AUC	23.5	21.31	23.32	34.39	31.94	22.98	24.19
HR-AUC/AUC (%)	100	100	59	100	100	42	43
**1/10^th^ MIC induced**	**GA16833+**	**GA41317+**	**GA16531+**	**GA17570+**	**GA17457+**		
AUC	52.18	53.13	54.87	53.13	52.91		
HR-AUC	12.48	12.25	6.86	6.72	6.57		
HR-AUC/AUC (%)	24	23	12	13	12		

### 14- and 15-membered ring macrolide induction of *mef*(*E*)/mel eliminates heteroresistance, but noninducing macrolides do not affect heteroresistance.

**FIG 4:**
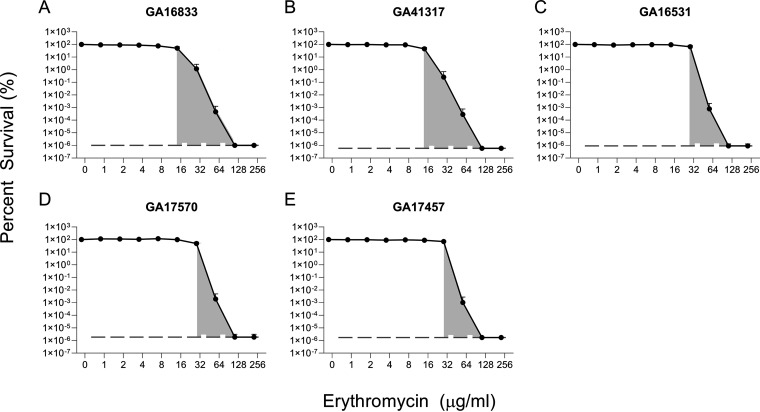
Erythromycin (14-member ring macrolide) induction of *mef*(E)/*mel* expression eliminates heteroresistance. Erythromycin PAP analyses were performed in triplicate for (A) GA16833, (B) GA41317, (C) GA16531, (D) GA17570, and (E) GA17457. The lower limit of detection is indicated on each graph with a dashed dark gray line. Shaded gray areas indicate the region from the highest noninhibitory concentration to the lowest concentration exhibiting maximal inhibition.

To determine the effect of increasing *mef*(E)/*mel* expression in a population-wide manner, the inducibility of the *mef*(E)/*mel* genes was exploited. As the Mega system is inducible by subinhibitory levels of erythromycin, five Mega-containing strains (MICs 2 to 12 μg/mL) were first induced and subjected to PAP analysis to determine if heteroresistance was maintained upon induction. High-level resistant strains GA16242 and GA71819 were not queried, as induction of these strains leads to MICs approaching 256 μg/mL, which is the highest concentration of erythromycin queried for the PAP analysis. After 1 h of induction with 0.1 μg/mL erythromycin, MICs were markedly elevated (MICs 16 to 64 μg/mL), and none of the strains now displayed heteroresistance, as there was less than a 4-fold difference between the lowest concentration exhibiting maximal inhibition and the highest noninhibitory concentration (Fig. 4a to e). With erythromycin induction, the HR-AUC values (gray shading, Fig. 4, Table 4) falling beyond the MIC breakpoints ranged from 6.57 to 12.48 and were 12 to 24% of the total AUC (Table 4).

**FIG 5:**
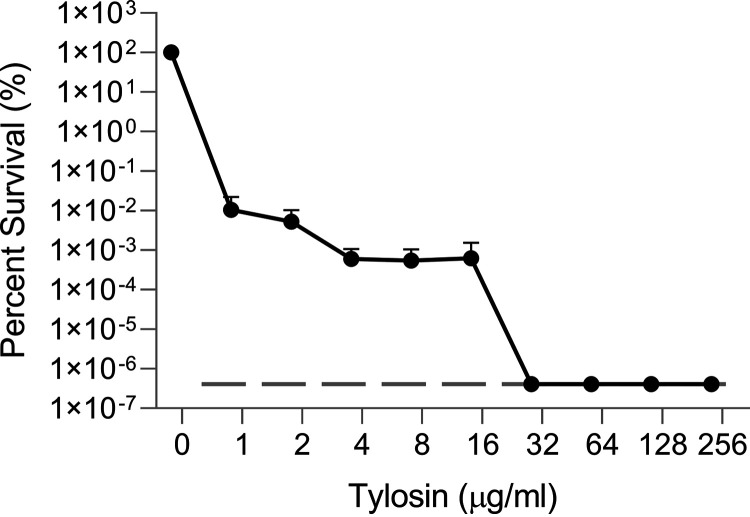
Treatment with a noninducing 16-member ring macrolide, tylosin, does not alter the heteroresistance phenotype. GA17457 was treated with a subinhibitory concentration of tylosin for 1 h before PAP analysis was performed on erythromycin-containing plates. The lower limit of detection is indicated on the graph with a dashed dark gray line.

While the *mef*(E)/*mel* operon is induced by most macrolides containing 14- and 15-membered rings, other macrolides such as the 16-membered ring macrolide tylosin do not induce expression (11). To determine the effect of a non-*mef*(E)/*mel*-inducing macrolide such as tylosin on the erythromycin resistance phenotype, GA17457 was incubated with subinhibitory concentration of tylosin for 1 h before a PAP analysis was performed. Incubation with tylosin did not alter the heteroresistance patterns seen in GA17457 (Fig. 5), supporting the hypothesis that the induction of *mef*(E) and *mel* is required for heteroresistance.

### The *mef*(E)*L* leader peptide sequence of the 5′ regulatory region is required for heteroresistance and induction.

**FIG 6:**
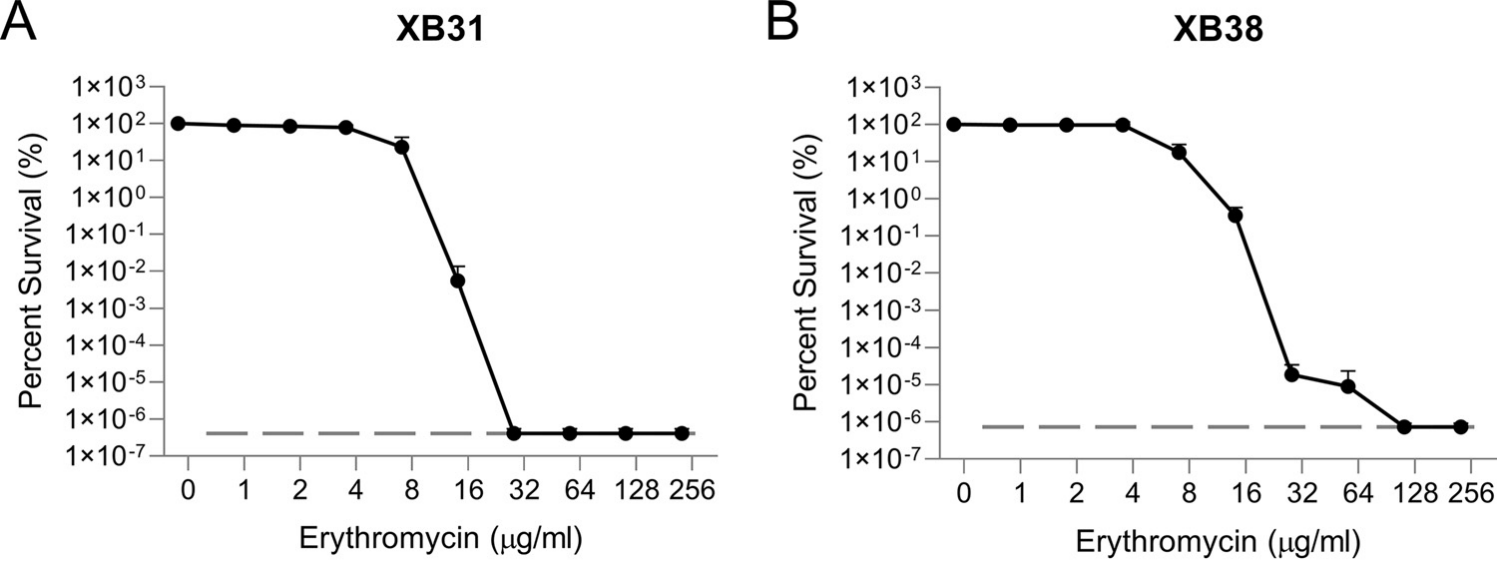
Deleting +19-298 nucleotide sequence of the 5′ regulatory region of *mef*(E)/Mega removes heteroresistance, and restoring the *mef*(E)*L* sequence restores heteroresistance. Mutant XB31 contains a deletion of +19-298 of the 5′ regulatory region. Mutant XB38 retains the *mef*(E) leader peptide sequences +19-62. Mutations are in the GA17457 background. Erythromycin PAP analyses were performed in triplicate for (A) XB31, and (B) XB38. The lower limit of detection is indicated on each graph with a dashed dark gray line.

To further explore the relationship between inducibility and heteroresistance, a 5′ regulatory region deletion mutant XB31, which was unable to be induced, was used (Table 3). This mutant is still able to express *mef*(E)/*mel* at low levels and provide resistance at similar levels to the wild-type uninduced parent strain GA17457. When induced with erythromycin, XB31 MICs are increased less than 2-fold, unlike the 2-to-4-fold increase in erythromycin MICs seen in the parent strain (Table 3). To determine if this strain displayed heteroresistance, a PAP analysis was performed. XB31 displayed traditional resistance patterns indicative of a homogeneously resistant population (Fig. 6A).

**FIG 7:**
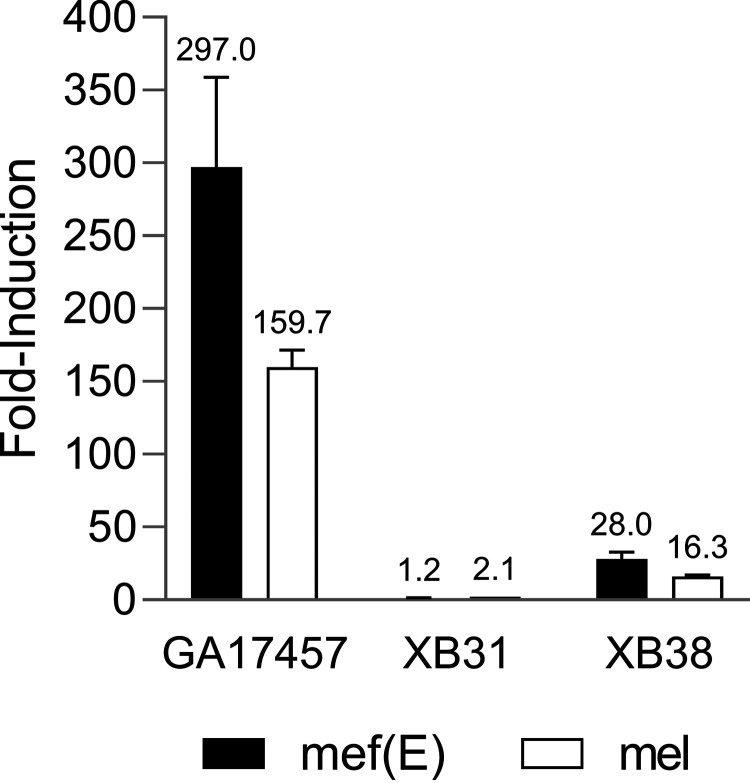
The *mef*(E) leader peptide sequence is required for induction of *mef*(E) and *mel* transcripts. *mef*(E) and *mel* expression levels were determined in wild-type GA17457, a *mef*(E)/*mel* 5′ regulatory region deletion mutant (XB31), and a *mef*(E) leader peptide-containing 5′ regulatory region deletion mutant (XB38) relative to the housekeeping gene *fab*(K), and the values of uninduced were compared to the induced mRNA levels. The numbers above the bars indicate the fold induction value average over three replicates.

As previously shown (22), XB38, a deletion mutant of the 5′ regulatory region apart from the *mef*(E)*L* peptide-encoding sequence, retains the ability to be strongly induced by subinhibitory concentrations of erythromycin (Table 3). To see if the XB38 mutant was also heteroresistant, a PAP analysis was performed. The presence of the *mef*(E)*L* sequence restores a heteroresistant phenotype (Fig. 6B). To confirm that the induction of *mef*(E)/*mel* at a transcriptional level followed heteroresistance patterns, the transcript levels for *mef*(E) and *mel* were queried in wild-type GA17457 and mutants XB31 and XB38. The uninducible XB31 showed no significant increase in either *mef*(E) or *mel* transcript levels (Fig. 7). This is in contrast to wild-type GA17457 and XB38. The fold induction in the XB38 mutant was reduced relative to the wild type, likely attributable to the persistent deletion of 235 bp of the operon’s promoter. Both GA17457 and XB38, however, showed significant increases in *mef*(E) and *mel* transcript levels (Fig. 7).

## DISCUSSION

The Mega element in *Spn* results in a broad range of observed MICs to macrolides and has been well characterized by genetic structure, gene products, genomic locations, and mechanism of inducibility by macrolides (8, 9, 11). This study links *mef*(E) and *mel* expression levels and MIC and documents heteroresistance associated with the Mega-mediated macrolide resistance phenotype in *Spn*. The heteroresistance phenotype spans Mega-type, Mega-insertion class, and high and low levels of macrolide resistance, and is dependent upon a *Spn* strain’s ability to express *mef*(E)and *mel*.

**FIG 8:**
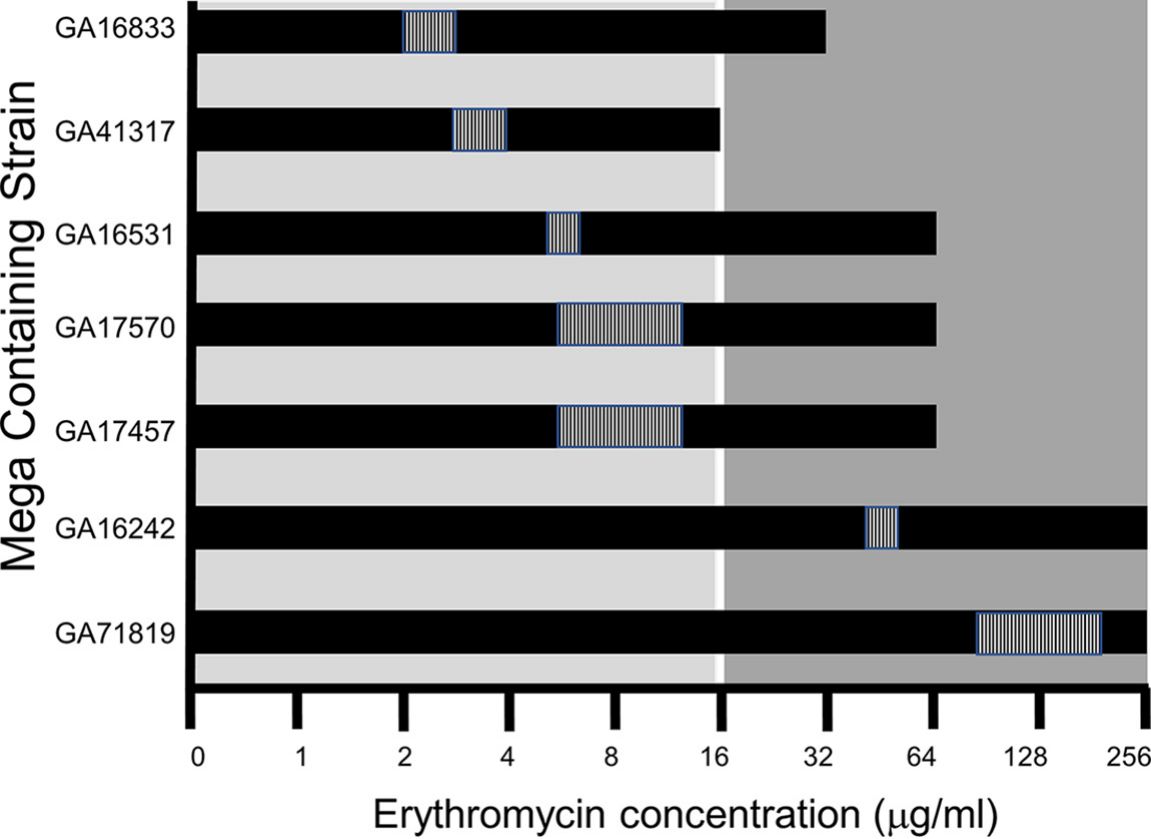
All Mega-containing strains contain heteroresistant populations which are resistant to high levels (16 μg) of erythromycin. For representative strains which encompass the full range of *mef*(E) and *mel* associated MICs, the range of concentrations of erythromycin in which populations within the strain can grow are shown with black bars. The light gray box indicates clinically defined low levels of macrolides, the dark gray box indicates clinically defined high levels of macrolides, and the white line indicates the 16 μg/mL cutoff for high-level resistance. The hatching within the black bars indicates the Etest-determined MIC values.

A range of Mega-containing *Spn* strains were queried in this study; all were found to contain subpopulations that can persist in the face of high-level macrolides (Fig. 8). All examined strains are characterized as resistant to macrolides (MIC >1 μg/mL); however, Fig. 8 demonstrates that all strains reach or surpass the breakpoint of 16 μg/mL used as a cutoff for high-level macrolide resistance (23). As *Spn* is a naturally transformable organism, even more so in the native environment of the NP (24), this is concerning clinically for the spread of resistance mechanisms that could lead to high-level resistance, as could occur in instances of *Spn* strain cocolonization of the NP (25).

Macrolide heteroresistance and the inducibility of the *mef*(E)/*mel* operon are linked in *Spn* containing the Mega element. More broadly, it is hypothesized that inducible systems are likely to demonstrate antibiotic heteroresistance, which will require future studies. As previously described, the mechanism of macrolide inducibility is dependent on ribosome stalling at the *mef*(E)L peptide sequence, facilitating stability of the mRNA (22). For mutant XB31, in which the leader peptide sequence has been deleted, there is no operon induction, and in strains in which expression is upregulated via induction, heteroresistance is eliminated. The stochastic induction of *mef*(E) and *mel* in a subpopulation leads to increased resistance to macrolides in the inducible Mega-containing strains. This mechanism resembles the stochastic expression in the antibiotic resistance activator *mar*(A) in E. coli, resulting in heteroresistance (26). The heteroresistant colonies isolated from Mega-containing strains display the same resistance phenotype as the parent strains, and are thus not a result of a gene mutation or gene-amplification. Highly resistant subpopulations resulting from gene mutations or gene amplifications have stable or semistable expression of higher levels of resistance than their parental strains (18, 27). Selection for heteroresistant colonies using high concentrations of antibiotic also confirmed that there were no alterations in gene copy number of either *mef*(E) or *mel*.

Heterogeneity in gene expression is advantageous when populations of bacteria are exposed to environmental fluctuations, including antibiotic selection (28, 29). Heterogeneity is typically ascribed to stochastic gene expression, or cell-to-cell fluctuations in expression levels (30). Stochastic expression of an antibiotic resistance gene activator provides transient resistance in single cells in the *mar*(A) system (26). Transiently resistant phoenix colonies of Pseudomonas aeruginosa grow within the zone of inhibition of aminoglycoside antibiotics, but numbers of colonies which emerge remain stable from generation to generation, indicating no heritability of high levels of resistance (31), similar to the phenotype seen with Mega-associated heteroresistance, pointing to a potential similar stochastic gene expression mechanism. Such fluctuations in gene expression in the macrolide-resistance Mega system can induce resistance in the subpopulation of cells which have upregulated *mef*(E) and *mel*. This study provides evidence for the hypothesis put forward by Nicoloff et al., in which stochastic gene expression can lead to possible heteroresistance (18).

Macrolide treatment failures in lower respiratory infections and bacteremia are not uncommon (32 to 35). Gonzalez et al. documented macrolide (azithromycin) treatment failures, in which 8/11 successfully isolated strains demonstrated the M phenotype (20). Half of the M-phenotype strains for which MIC data were available were associated with high-level macrolide resistant MICs, and the other half were not (20). Other studies have also shown treatment failures resulting from strains demonstrating the M phenotype (21) or the presence of *mef* more specifically (33). This is further supported by data presented in a population-based surveillance study of cases of pneumococcal bacteremia, which found that low-level resistance conferred by *mef*(A) was found to be overrepresented among macrolide failures (36). Heteroresistance associated with the Mega element may contribute to these treatment failures observed with macrolides.

Heteroresistant populations can be successfully treated by combination therapies, as subpopulations which are resistant to one antibiotic are generally independent from those that are resistant to a second antibiotic (37). Combination therapy is already used in the treatment of community-acquired pneumonia in the cases of presence of comorbidities or risk factors for drug-resistant *Spn* infection, and treatment with a beta-lactam or tetracycline plus a macrolide is often recommended in these cases (5, 38). Our data suggest any Mega-containing *Spn* strains are capable of heteroresistance and should be considered high level when making clinical treatment decisions.

Heteroresistance is often undetected due to the low frequency of resistant subpopulations, on the order of 1 in 10,000 cells (39). Whole-genome sequencing approaches are enhancing antibiotic resistance predications (40), and whole-genome sequencing is used in monitoring of invasive *Spn* isolates (41). Understanding the mechanisms underlying heteroresistance allows more accurate predictions of antibiotic resistance based on genomic data. This study adds to the understanding of heteroresistance in Mega-containing *Spn* isolates.

## MATERIALS AND METHODS

### Strains and growth conditions.

Representative strains were chosen to encompass the full range of *mef*(E)/*mel* associated MICs and are listed in Table 1. XB30, XB31, and XB38 were mutants made in the GA17457 background and detailed in Chancey et al., 2015 (22). *Spn* strains were grown on Trypticase soy agar (TSA) II containing 5% sheep’s blood (blood agar) or in Todd-Hewitt broth containing 0.5% yeast extract broth (THY). Plate cultures were grown at 37°C with 5% CO_2_, and broth cultures were grown in a 37°C water bath.

### Antibiotic susceptibility.

*Spn* strains were streaked from frozen onto TSAII blood agar plates with or without 0.1 μg/mL erythromycin and grown for 18 to 20 h (11, 32). Bacteria from these plates were resuspended in cation-adjusted Mueller-Hinton broth to an optical density of 0.5 McFarland standards. These suspensions were streaked onto Mueller-Hinton agar containing 5% sheep’s blood, and erythromycin Etest strips (bioMérieux) were applied. The plates were incubated for 20 to 24 h before reading results.

### Population analysis profiling.

A modified protocol of the microdilution plating method for population analysis profiling (42) was performed as detailed below. *Spn* strains were streaked from frozen onto blood agar plates and grown overnight. Primary THY cultures were inoculated to an OD600 of approximately 0.2, and grown for 90 to 120 min. Secondary cultures were inoculated at an OD600 of 0.05 and allowed to grow to early midlog phase (OD600 ~0.3) before half the culture was treated with 1/10th MIC of erythromycin (or tylosin, as indicated) for an hour before both untreated and treated cultures were serially diluted in cation-adjusted Mueller-Hinton broth. Thirty microliters of −1, −3, and −5 dilutions were spotted in triplicate on Mueller-Hinton agar plates with 5% sheep’s blood and 2-fold increasing concentrations of antibiotics. Plates were incubated for 20 to 24 h before colonies were counted. The following concentrations of antibiotic were used for erythromycin and tylosin: 0, 1, 2, 4, 8, 16, 32, 64, 128, 256 μg/mL. For AUC calculations, log-transformed values for percent surviving bacteria were plotted versus the number of dilutions tested. GraphPad Prism version 9.1.0 for Windows (GraphPad Software, San Diego, California, USA) was used to calculate areas under the curve (AUC).

### qPCR.

A modified protocol for qPCR was performed based on previously published methods (43). *Spn* strains were streaked and grown through secondary culture as above, grown in triplicate. One culture remained untreated, one was treated with 1/10th MIC, and the final culture was treated with 192 μg/mL erythromycin. Cultures were grown for 1 h before the bacteria were pelleted at 4K rpm for 5 min and frozen at −80°C. Genomic DNA was isolated from the pellets using the Zymo Quick-DNA Fungal/Bacterial Miniprep kit (Zymo Research) according to the manufacturer’s protocol. Genomic DNA concentration was determined using a NanoDrop 8000 spectrophotometer. The samples were diluted to 15 ng/μL, and qPCR was performed with standard curves using 10-fold serial dilutions of untreated genomic DNA. Gene copy numbers were determined relative to the *fab*(K) control using the ΔCt method.

### qRT-PCR.

*Spn* strains were streaked from frozen onto blood agar plates and grown overnight. Primary THY cultures were inoculated to an OD600 of approximately 0.2 and grown for 90 to 120 min. Secondary cultures were inoculated at an OD600 of 0.05 and allowed to grow to midlog phase. Half of the culture was treated with 1/10th MIC of erythromycin for an hour before both untreated and treated cells were collected and treated with RNAprotect Bacterial Reagent (Qiagen). RNA was isolated using the RNeasy minikit (Qiagen) and subjected to DNA-free DNase treatment (Invitrogen) before cleanup with an RNA clean and concentrator kit (Zymo Research). RNA was quantitated, and equal amounts of RNA from each sample (150 ng) were used to create cDNA libraries using the iScript Reverse Transcription Supermix kit (Bio-Rad). cDNA was diluted 1.5× before qRT-PCR. qRT-PCR was performed using iQ SYBR green Supermix (Bio-Rad) with a CFX96 real-time PCR detection system. qRT-PCR primers used are listed in Table A1 in the supplemental material. Expression values were calculated using the ΔΔC_T_ method.
