# Supplementary material for: Inducible Mega-Mediated Macrolide Resistance Confers Heteroresistance in Streptococcus pneumoniae
Source: Antimicrob Agents Chemother. 2023 Feb 27;67(3):e01319-22. doi: 10.1128/aac.01319-22 (PMC10019249; doi:10.1128/aac.01319-22)
Supplement: Supplemental file 1 — Supplemental material. Download aac.01319-22-s0001.pdf, PDF file, 0.1 MB [file aac.01319-22-s0001.pdf]

## Supplemental Materials

**Supplemental Table A1.** Primers used for qRT-PCR and qPCR.

| Primer Name | Primer Sequence | Primer Target | Source |
|---|---|---|---|
| qfabK F1 | TCGGTGCAGACGCTGTTATT | *fab*(K) housekeeping gene | This publication |
| qfabK R1 | GCAATTCCTCCTGCAGCAAT | *fab*(K) housekeeping gene | This publication |
| qmef_F3 | GTATTCCCGAAACGGCTAAACTG | *mef*(*E*) | Schroeder et al., 2019 (1) |
| qmef_R3 | TGGAACGCCTGTGCATATTTC | *mef*(*E*) | Schroeder et al., 2019 (1) |
| qmel_F2 | TTCTGCACCGACTATAGGGTATGG | *mel* | Schroeder et al., 2019 (1) |
| qmel_R2 | AAACCCTAGAGCACAGGATTGC | *mel* | Schroeder et al., 2019 (1) |

## Supplemental References


1. Schroeder MR, Lohsen S, Chancey ST, Stephens DS. 2019. High-Level Macrolide Resistance Due to the Mega Element [mef(E)/mel] in Streptococcus pneumoniae. Front Microbiol 10:868.
